# Cytotoxic Lesions of the Corpus Callosum (CLOCC) Suggesting Exacerbation by Heterogeneous COVID-19 Booster Vaccination

**DOI:** 10.7759/cureus.41105

**Published:** 2023-06-28

**Authors:** Yuta Chiba, Yoshiaki Takahashi, Rie Kawakita, Kazushi Deguchi, Tsutomu Masaki

**Affiliations:** 1 Gastroenterology and Neurology, Kagawa University, Kita-gun, JPN; 2 Neurology, Kagawa Prefectural Central Hospital, Takamatsu, JPN

**Keywords:** covid-19, corpus callosum, reversible splenial lesion, mild encephalopathy/aseptic encephalitis, interleukin-6, ataxia syndrome, vaccine adverse reactions

## Abstract

Cytotoxic lesions of the corpus callosum (CLOCC) is a disease entity associated with reversible lesions of the corpus callosum on magnetic resonance imaging (MRI). CLOCC is caused by a variety of etiologies, but CLOCC after vaccination is extremely rare. Four prior cases of CLOCC after the first dose of severe acute respiratory syndrome coronavirus 2 (SARS-CoV-2) mRNA vaccine have been reported; these were localized to the splenium and showed early clinical and neuroradiological recovery. We experienced an unusual case in which a heterogeneous COVID-19 booster vaccination caused rather severe CLOCC damage.

A 74-year-old Japanese woman presented with ataxia, high fever, and hearing loss several days after her third vaccination against COVID-19. This booster was an mRNA-1273 while her first and second vaccinations were both BNT162b2 type. SARS-CoV-2 real-time reverse transcriptase polymerase chain reaction (RT-PCR) analysis was negative, but serum SARS-CoV-2 S-IgG antibodies were elevated. Her cerebrospinal fluid (CSF) showed an elevated cell count and high levels of protein and interleukin-6 (IL-6). Brain MRI showed CLOCC spreading throughout the body of the corpus callosum. After the exclusion of other potential causes, the diagnosis of vaccination-related CLOCC was made. Six months later, recovery of clinical and MRI findings remained incomplete. It was suggested that the patient's CLOCC might have been caused by the increase in CSF IL-6 due to an enhanced immune response from the heterogeneous vaccination, resulting in more severe damage to the corpus callosum than usual.

## Introduction

Cytotoxic lesions of the corpus callosum (CLOCC), formerly known as mild encephalopathy with reversible splenial lesions (MERS), is a disease entity associated with reversible lesions of the corpus callosum on magnetic resonance imaging (MRI). Reported etiologies of CLOCC include viral or other types of infectious pathogens, epilepsy and antiepileptic drug use, metabolic diseases, drug-related toxicity, malignancies, cerebrovascular diseases, traumatic brain injury, status migrainosus, and high-altitude disease [1]. After the recent pandemic outbreak, COVID-19 was added to the list of viruses potentially causing CLOCC [2]. Although four cases of CLOCC as a sequela to mRNA vaccines against severe acute respiratory syndrome coronavirus 2 (SARS-CoV-2) have been reported, all four developed CLOCC localized to the splenium after the first dose of SARS-CoV-2 mRNA vaccine, with early clinical and neuroradiological recovery, as is typical of CLOCC [1,3-5]. We report a case of an elderly woman presenting with severe, widespread CLOCC a few days after she received a COVID-19 vaccine booster (mRNA-1273) that differed from the first and second vaccines she had received (both BNT162b2). To our knowledge, this is the first report associating heterogeneous vaccination against SARS-CoV-2 mRNA with a worse prognosis of CLOCC.

## Case presentation

A 74-year-old right-handed Japanese woman with an unstable gait due to an ankle deformity but who was living independently in her daily life had received two prior COVID-19 vaccines (both BNT162b2) without any adverse reaction. A few hours after a third dose of the COVID-19 vaccine (mRNA-1273) (day 1), she developed chills, general malaise, and anorexia. On the 5th day, she fell several times and experienced writing difficulties. On the 7th day, she was admitted to a local hospital due to the onset of a 39.2°C fever and hearing loss. At admission, her body mass index was 17.8 and her serum albumin was 2.3 mg/dL, suggesting poor nutrition. The result of a SARS-CoV-2 real-time reverse transcriptase polymerase chain reaction (RT-PCR) analysis of a nasal swab was negative. Chest CT showed no abnormalities. A bacterial infection was suspected based on a white blood cell count of 11700/μL and C-reactive protein (CRP) at 10.33 mg/dL, and cefotaxime and levofloxacin were administered. However, ambulation became more difficult. The laboratory findings remained unchanged. On the 15th day, the patient was referred to our hospital.

She had a persistent fever in the 39°C range. There was no chondromalacia or blistering of the auricle. Severe sensorineural hearing loss (SSHL) with a mean hearing of 102.5 dB on both sides was observed with pure tone audiometry. Muscle strength in the extremities was almost normal. Muscle tonus was normal. Tendon reflexes were normal bilaterally. Both plantar responses were flexor. She showed intention tremor of the right upper limb and difficulty maintaining a sitting position due to severe truncal ataxia. No apraxia or agraphia of the left hand suggestive of disconnection syndrome was observed. Routine blood test results were unremarkable other than neutrophilic leukocytosis (white blood cells: 8800 with 91.9% neutrophils), a high CRP level at 16.5 mg/mL, and albumin at 2.1 mg/dL.

Test results were negative for markers of autoimmune disease: antinuclear antibody, anti-double stranded (ds) DNA antibody, anti-SSA antibody, anti-SSB antibody, myeloperoxidase-antineutrophilic cytoplasmic antibody (MPO-ANCA), proteinase3-antineutrophilic cytoplasmic antibody (PR3-ANCA), anti-GM1 antibody, anti-GQ1b antibody, anti-myelin oligodendrocyte glycoprotein (MOG) antibody, anti-aquaporin (AQP) 4 antibody, anti-glial fibrillary acidic protein (GFAP)-α antibody, and paraneoplastic neurological syndrome-related antibodies. Serum angiotensin-converting enzyme (ACE) was within normal limits. Serum IgM for mumps and cytomegalovirus was negative. Cerebrospinal fluid (CSF) showed a cell count at 49/μL, protein at 95 mg/dL, and interleukin-6 (IL-6) at 633 pg/mL (cut-off value <7 pg/mL). In the CSF, IgM and RT-PCR for varicella-zoster virus (VZV) were negative. The CSF cytology was class I. There was no elevation of soluble interleukin-2 receptor (sIL-2R) in the patient's serum or CSF. Blood and CSF cultures showed no evidence of bacterial growth.

**Figure 1:**
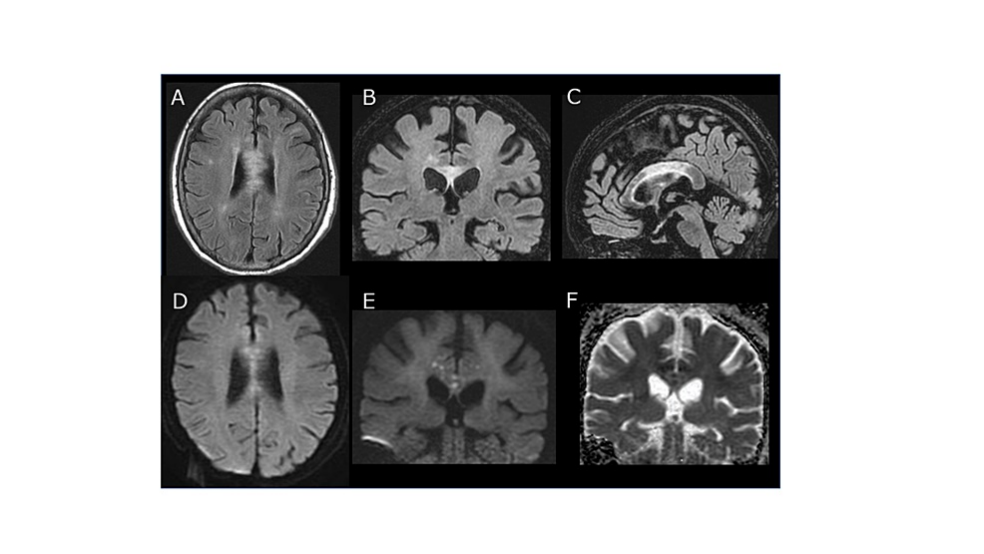
The patient's first brain MRI on the 19th day A–C: FLAIR image showed hyperintensity over the body of the corpus callosum. Hyperintensity on a DWI (D,E) and low ADC values (F) were noted scattered in dots, suggesting CLOCC. FLAIR, fluid-attenuated inversion recovery; DWI, diffusion-weighted image; ADC, apparent diffusion coefficient; CLOCC, cytotoxic lesions of the corpus callosum

On the 19th day, diffusion-weighted image (DWI) and fluid-attenuated inversion recovery (FLAIR) on brain MRI demonstrated high-intensity signals spreading throughout the body of the corpus callosum. The same area showed a reduced apparent diffusion coefficient (ADC) (Figure 1). Gadolinium enhancements on T1-weighted imaging were not observed. An examination with an infrared charge-coupled device (CCD) camera showed no nystagmus.

**Figure 2:**
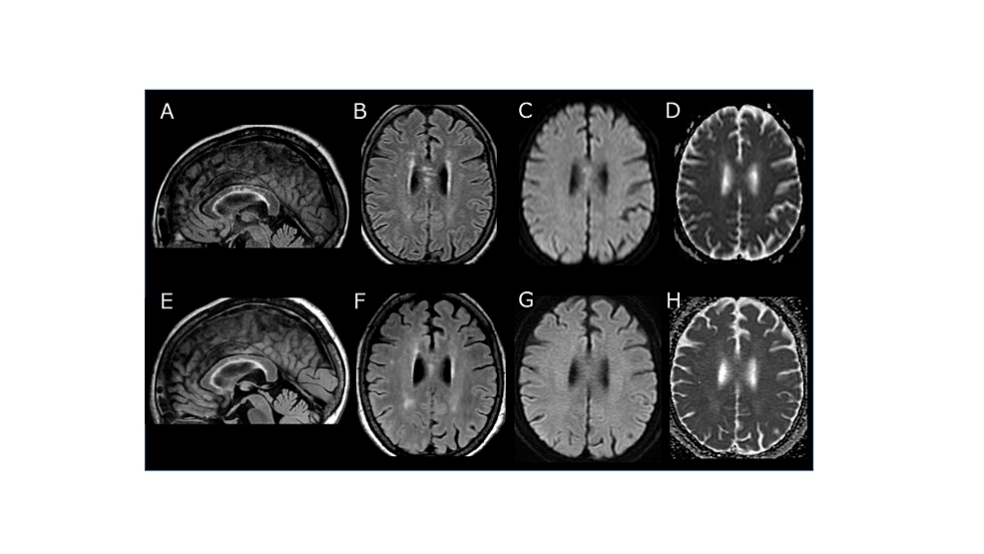
Follow-up brain MRI on the 70th day (A–D) and 207th day (E–H) Hyperintensity on FLAIR imaging was reduced on the 70th day (A,B) but still remained on the 207th day (E,F). Hyperintensity on DWI and low ADC values were not noticeable on the seventieth day (C,D) and had completely disappeared by the 207th day (G,H). FLAIR, fluid-attenuated inversion recovery; DWI, diffusion-weighted image; ADC, apparent diffusion coefficient

Since meningoencephalitis was suspected, cefotaxime (2 g/day four times daily), ampicillin (2 g/day four times daily), vancomycin (1 g twice daily), acyclovir (10 mg/kg three times daily), and dexamethasone (6.6 mg four times daily) were administered as empiric therapy. However, the patient's drowsy state and fever continued for several days. On the 30th day, she was able to take a few steps with a walker. On the 47th day, the CSF showed a cell count of 5/μL, protein level of 63 mg/dL, and IL-6 level of 2.3 pg/mL. SARS-Cov-2 S-IgG showed marked elevation at 4990 AU/mL (cut-off index <1 AU/mL). On the 70th day, FLAIR on brain MRI showed that the corpus callosum lesions had diminished but not disappeared; neither hyperintensity on DWI nor low ADC values were noted (Figure 2).

By the seventy 6th day, the patient's intention tremor of the right upper limb had almost disappeared, and she was able to maintain a sitting position and walk around the ward with a walker. Her hearing loss was alleviated, averaging 61.3 dB on the right side and 60.0 dB on the left side over the entire frequency range, but did not recover further thereafter. As a result of intensive rehabilitation, after six months the patient was able to walk with a broad-based gait using a typical walker, but she had not recovered to her premorbid condition. On the 207th day, FLAIR imaging showed a slight residual lesion in the corpus callosum (Figure 2).

## Discussion

The patient developed a high fever, drowsiness, hearing loss, and ataxia with CLOCC after receiving a third SARS-Cov-2 mRNA vaccination of a type that differed from those of her previous vaccinations. Her case differed from the usual course of CLOCC, in which clinical and MRI findings recover completely within a short period of time [1]; her recovery took more time and remained incomplete.

It has been reported that various central nervous system disorders and peripheral neuropathy might occur approx. 7-11 days after an individual's first or second COVID-19 vaccination [6-8]. The suggested mechanisms of these complications have included molecular mimicry, aberrant immune reaction, and neurotoxicity [9,10]. Four cases of CLOCC localized to the splenium caused by a first dose of the SARS-CoV-2 mRNA vaccine (BNT162b) have also been reported [3-5]. Our patient's case was diagnosed as CLOCC associated with a booster SARS-CoV-2 mRNA vaccine (mRNA-1273) because (i) no known factors causing CLOCC were identified other than the mRNA-1273 vaccine, and (ii) the patient developed neurological symptoms with elevated SARS-Cov-2 S-IgG antibody 5 days after the vaccination [1].

Although clinical and MRI findings of CLOCC due to COVID-19 vaccination have shown good recovery by 2 months after onset, the present elderly patient with physical frailty obtained only an incomplete recovery even after 6 months [3-5]. Muscle strength remained normal during the clinical course, suggesting that the prolonged recovery was not simply due to rest-induced sarcopenia. The precipitating mRNA vaccine was her third: the first and second doses were BNT162b2 and the third dose was mRNA-1273. Heterogeneous vaccinations have been reported to result in higher antibody titers (mean value, 29,646U/mL after 1 month of third vaccination) than that of additional vaccinations with the same vaccine (mean value, 19,865U/mL after 1 month of third vaccination) [11]. Therefore, a highly enhanced immune response might have caused the more severe neurological and MRI findings in this patient.

It has been suggested that acute disseminated encephalomyelitis (ADEM) and CLOCC may be diseases of the same spectrum [12]. Similar to ADEM, which can occur after vaccination, a small number of cases of CLOCC have been reported after mumps vaccination [13]. Given this case and prior ones, COVID-19 vaccination should be listed as one of the etiologic agents of CLOCC. In COVID-19, inflammatory processes involving cytokines such as IL-6 may trigger an accumulation of glutamate in the extracellular space and cause cytotoxic edema in the corpus callosum, where cytokines and glutamate receptors are densely expressed [1,14]. Since the present patient's CSF showed a marked increase in IL-6, the involvement of a cytokine storm similar to COVID-19 might have caused severe cytokine-induced injury to the corpus callosum. In addition, genetic variants in Toll-like receptor 3 (although not confirmed in this case), which is important for the recognition of foreign RNA, might affect the risk of adverse events from an mRNA vaccine [15].

Although hearing loss has been reported to occur 3.26 times per million doses of the COVID-19 vaccine [6], the incidence of sudden sensorineural hearing loss (SSHL) after COVID-19 vaccination does not statistically exceed that of the general population [16]. The relationship between SSHL and COVID-19 vaccinations is thus unclear [16,17]. It has been speculated that if there is a causal relationship between the two, SSHL due to VZV reactivation after COVID-19 vaccination, the involvement of molecular mimicry between the structure of the vaccine adjuvant and the specific human epitope [18], and/or cochlear ischemia caused by thrombosis or vasospasm of the inner ear artery [19] may be involved. However, in the present case, VZV had not been reactivated, based on the patient's clinical findings, serum and CSF IgM and the CSF RT-PCR result for VZV. An increased risk of thrombosis has been noted only with viral vector vaccines [20], which does not apply to the present case in which an mRNA vaccine was used.

## Conclusions

As a result of the heterogeneous booster COVID-19 vaccination, our patient developed ataxia due to CLOCC and experienced SSHL. These symptoms did not fully recover, unlike previous COVID-19 vaccination-related cases. The enhanced immune response by heterogeneous vaccination might have caused severe CLOCC through the actions of cytokines and glutamate. The association between COVID-19 vaccines and hearing loss is not clear at this time.
